# Effects of eating with an augmented fork with vibrotactile feedback on eating rate and body weight: a randomized controlled trial

**DOI:** 10.1186/s12966-019-0857-7

**Published:** 2019-10-22

**Authors:** Sander Hermsen, Monica Mars, Suzanne Higgs, Jeana H. Frost, Roel C. J. Hermans

**Affiliations:** 1 Utrecht University of Applied Sciences, Utrecht, NL Netherlands; 2 Division of Human Nutrition, Wageningen University, Wageningen, NL Netherlands; 3 School of Psychology, University of Birmingham, Birmingham, UK; 4 VU University Amsterdam, Amsterdam, NL Netherlands; 5 NUTRIM School of Nutrition and Translational Research in Metabolism, Maastricht University, PO BOX 616, 6200 MD Maastricht, The Netherlands; 6 Behavioural Science Institute, Radboud University, Nijmegen, NL Netherlands

**Keywords:** Eating rate, Weight loss, Randomized controlled trial, Feedback, Sensory

## Abstract

**Background:**

Eating rate is a basic determinant of appetite regulation: people who eat more slowly feel sated earlier and eat less. A high eating rate contributes to overeating and potentially to weight gain. Previous studies showed that an augmented fork that delivers real-time feedback on eating rate is a potentially effective intervention to decrease eating rate in naturalistic settings. This study assessed the impact of using the augmented fork during a 15-week period on eating rate and body weight.

**Methods:**

In a parallel randomized controlled trial, 141 participants with overweight (age: 49.2 ± 12.3 y; BMI: 31.5 ± 4.48 kg/m2) were randomized to intervention groups (VFC, *n* = 51 or VFC+, *n* = 44) or control group (NFC, *n* = 46). First, we measured bite rate and success ratio on five consecutive days with the augmented fork without feedback (T1). The intervention groups (VFC, VFC+) then used the same fork, but now received vibrotactile feedback when they ate more than one bite per 10 s. Participants in VFC+ had additional access to a web portal with visual feedback. In the control group (NFC), participants ate with the fork without either feedback. The intervention period lasted four weeks, followed by a week of measurements only (T2) and another measurement week after eight weeks (T3). Body weight was assessed at T1, T2, and T3.

**Results:**

Participants in VFC and VFC+ had a lower bite rate (*p* < .01) and higher success ratio (*p* < .0001) than those in NFC at T2. This effect persisted at T3. In both intervention groups participants lost more weight than those in the control group at T2 (*p* < .02), with no rebound at T3.

**Conclusions:**

The findings of this study indicate that an augmented fork with vibrotactile feedback is a viable tool to reduce eating rate in naturalistic settings. Further investigation may confirm that the augmented fork could support long-term weight loss strategies.

**Trial registration:**

The research reported in this manuscript was registered on 4 November 2015 in the Netherlands Trial Register with number NL5432 (https://www.trialregister.nl/trial/5432).

## Background

In recent decades, the prevalence of excessive body weight has increased rapidly in North-American and European countries, including The Netherlands [1]. In 2017, almost one out of two Dutch adults are considered to have overweight [2]. Although a variety of factors are associated with overweight, evidence shows that eating quickly is positively associated with excess body weight (see 3 for a review), whereas a lower eating rate is associated with feeling sated earlier, smaller meals and a lower energy intake [3, 4]. Therefore, a promising method to support weight loss strategies may lie in encouraging those who eat quickly to slow down.

A potential barrier for an individual to change his or her eating rate may be its highly automatic habitual nature. In adults, eating rate is consistently found to be a personal characteristic, not dependent on context [5]. In addition, research suggests that eating rate also has a heritable component [6]. Recent technological developments present new ways to measure and alter such highly automatic behaviors by automatically sensing behavior and providing feedback on undesired behaviors as they occur [7].

A new and promising tool to alter eating rate is an augmented fork that contains sensors and actuators that provide real-time vibrotactile feedback on eating rate; when users of the fork eat too fast (i.e., taking more than one bite per 10 s), they feel a gentle vibration in the handle of the fork. This real-time vibrotactile feedback encourages people to slow down as they eat. The data provided by the fork is also available as retrospective visual feedback through a secure online dashboard, which is known to increase motivation to sustainably perform a desired behavior [8]. Previous research has suggested that the fork is acceptable to users [9] and is capable of reducing eating rate during a single meal in a laboratory context [10]. However, the fork has not been tested outside the lab, nor has it been tested for periods longer than a single meal.

Therefore, the objective of the current work is to assess the impact of the intervention – a fork that provides feedback on bite frequency – on eating rate and body weight during a 15-week intervention period. Specifically, we aimed to examine the effects of two forms of feedback, i.e. vibrotactile feedback and, in addition, vibrotactile feedback plus retrospective visual feedback, on eating rate and body weight. To do so, we conducted a three-armed parallel Randomized Controlled Trial (RCT) with two measures of eating rate, that is bite rate (i.e., average number of bites per minute) and success ratio (i.e., the percentage of bites with a pause of at least ten seconds between them). Furthermore, body weight was assessed at three time points; at baseline (T1), directly after the 4-week intervention period (post-intervention, T2) and at a follow-up after eight weeks (T3). Based on the evidence regarding the effectiveness of feedback to disrupt habitual behavior [7] and our previous work on the efficacy of the augmented fork to decrease eating rate [9, 10], we hypothesized that frequent use of the augmented fork would lead to longer pauses and therefore a slower eating rate, which may translate to weight loss.

## Methods

### Participants

**Fig. 1:**
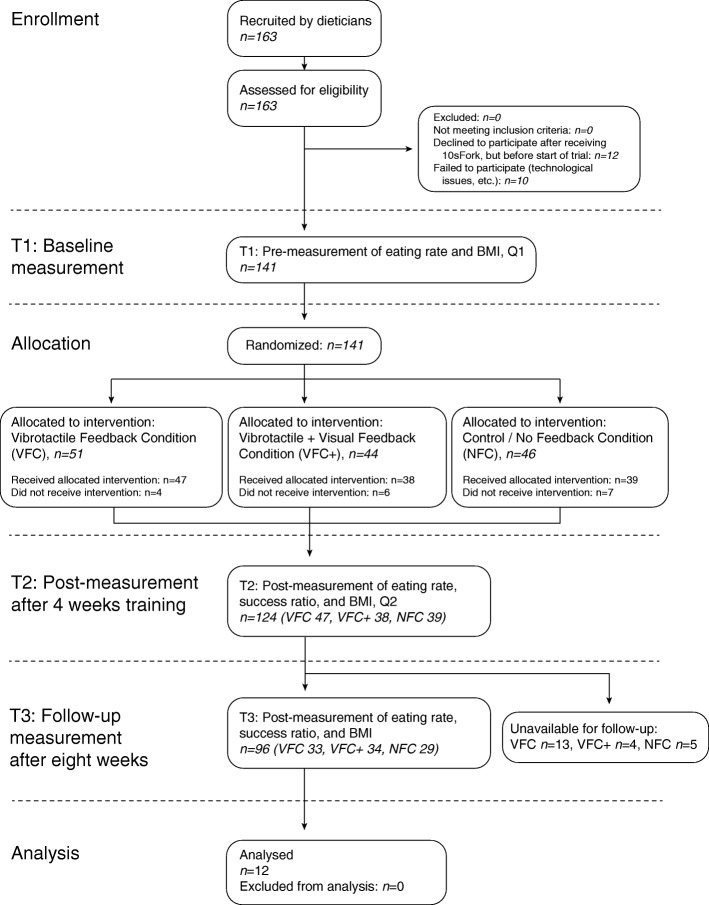
CONSORT flowchart of enrollment, allocation, and experimental design

Between November 2015 and April 2017, dieticians from 30 practices recruited participants from their practices who met the inclusion criteria for the current study: 1) participants were at least 18 years old, 2) participants were self-reported fast eaters (see Table 1) and 3) participants had a BMI score ≥ 25 kg/m^2^. Gastric bypass patients were excluded from the study. Our total sample consisted of 163 participants. Before volunteering, participants read a short leaflet about the study, which is available from the project’s OSF site. An overview of the enrollment process and the allocation to intervention conditions is available in Fig. 1 (CONSORT Flowchart).

**Table 1:** Baseline characteristics of study participants across three conditions^a,b^

Variable	NFC (*n* = 51)	VFC (*n* = 44)	VFC+ (*n* = 46)
Age, y *(SD)*	50.6 (11.0)	49.6 (11.0)	47.40 (14.4)
Female, n (% of questionnaire respondents)	21 (58% of 36)	26 (59% of 44)	22 (65% of 34)
BMI, kg/m^2^ *(SD)*	31.7 (5.2)	31.2 (4.4)	31.6 (3.9)
Perceived eating rate^c^ *(SD)*	7.7 (1.3)	7.6 (1.1)	7.7 (1.3)
Has eating discomfort^d^ (%)	3%	2%	0%
Has stomach complaints^d^ (%)	18 (47%)	26 (55%)	20 (53%)
Has diabetes type I^d^ (%), II^d^ (%)	3 (9%), 2 (6%)	2 (5%), 9 (21%)	5 (14%), 0 (0%)
Is on a diet^d^ (% of Q1 respondents)	10 (29%) of 36	15 (34%) of 44	15 (43%) of 34

^a^ – NFC, no feedback condition; VFC, vibrotactile feedback condition, VFC+, vibrotactile + visual feedback condition

^b^ – All data are for those participants allocated to the intervention (NFC *n* = 51; VFC, *n* = 44, VFC+, *n* = 46) AND filled out the premeasurement questionnaire (NFC *n* = 36; VFC, *n* = 44, VFC+, *n* = 34)

^c^ – On a scale from 1 to 10

^d^ – Dichotomous scale: Yes / No

The study was approved by the Ethics Committee of the Faculty of Social Sciences at Radboud University, Nijmegen, The Netherlands (ECSW2016–2501-363) ​and was in full accordance with the Helsinki Declaration of 1975 as revised in 2013. All procedures involved were preregistered in the Netherlands Trial Register with number NL5432. All participants provided their full written consent and received a gift voucher of €75 as compensation for participation.

### Design

**Fig. 2:**
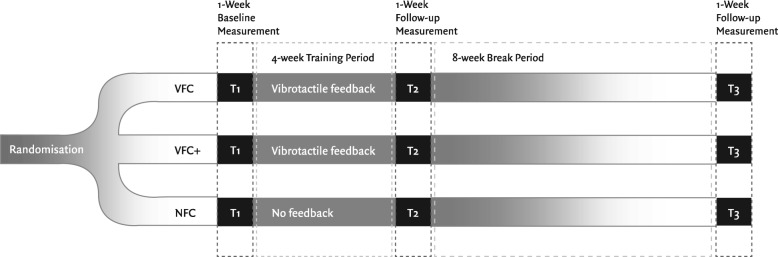
Flowchart of the experimental design of the study

The study was conducted between January 2016 and September 2017. The study was a three-armed parallel Randomized Controlled Trial (RCT) with three groups: 1) VFC: participants used an augmented fork for as many meals as possible in their home setting (and elsewhere if desired). When they ate quickly (pause less than 10 s between bites), the fork provided vibrotactile feedback. 2) VFC+, participants also received the vibrotactile feedback from the fork, but also had access to an online web portal with retrospective visual feedback on their eating rate and success ratio (the percentage of bites with a 10-s pause in between). And 3) NFC, participants ate with the augmented fork without any form of feedback. An overview of the design of the study is available in Fig. 2 (Design Flowchart).

The primary outcomes were two measures of eating rate, that is bite rate (average number of bites per minute), success ratio (the percentage of bites with a 10-second pause in between them), and body weight; these were measured at baseline (T1), directly after the 4-week intervention period (post-intervention, T2) and at a follow-up after eight weeks (T3). At T1 and T3 all participants ate with the augmented fork without any form of feedback for five consecutive days. Participants were randomized using simple randomization procedures (computerized random numbers list generated with an online randomizer tool [11]). Participants were assigned a number in order of enrollment in a single block; allocation was blinded to participants and their dieticians, we then assigned participants to 1 of three intervention groups.

### Smart fork intervention

After eligibility assessment, participants received the augmented fork, along with an instruction manual for the download and installation of the software needed for synchronization, a unique login code for the software, and instructions for fork use and maintenance. In addition, participants were briefed about how the fork measures eating rate. All instruction materials are available from the project’s OSF site. Participants were then invited by email to complete an online baseline survey, hosted on the Qualtrics platform, in which they provided information on their gender, age, health condition, motivation to change eating rate, perceived eating rate, perceived detriments of eating rate, perceived satiety, awareness of meal, and awareness of eating rate. Furthermore, participants were weighed and their height was measured by their dieticians, using standardized equipment and procedures at the dietician’s practice.

We then assessed participants’ eating rate during a baseline measurement (T1). All participants ate as many meals as possible with the fork during a period of five consecutive days. In this period, participants did not receive any form of feedback on their eating rate. Participants received no instructions on a minimum or maximum number of meals, or limitations on where they could use the fork, to minimize interference with their natural eating habits.

After establishing this baseline measure, we randomly assigned participants to one of three conditions. In all conditions, participants entered the intervention phase, consisting of a four-week period in which all participants were asked to eat as many meals as possible with the fork. Furthermore, participants in VFC and VFC+ received instructions about the way the fork provided vibrotactile feedback on eating rate. Additionally, participants in VFC+ received an invitation to visit an online website that provided visual retrospective feedback on their eating rate. This online dashboard was blocked for participants in NFC and VFC. During the four-week intervention, all participants ate as many meals as possible with the 10sFork.

After the intervention period, all participants entered a post-intervention measurement phase (T2), in which they used the fork without any form of feedback for five consecutive days, eating as many meals as possible with the fork to establish their post-intervention eating rate. Moreover, they were re-weighed by their dieticians.

Participants then entered a period of eight weeks in which they could not use the fork. After this period, in a follow-up measurement (T3), participants in all three conditions were once again re-weighed by their dieticians and used the fork without any form of feedback for five consecutive days, to test for sustainable changes in eating rate. Figure 2 (Design Flowchart) provides an overview of the procedure and experimental design of the study.

### Stimulus materials

Vibrotactile feedback on eating rate was provided by the 10sFork, developed and marketed by SlowControl (Paris, France). This augmented fork has the appearance of a regular fork but contains sensors and actuators that provide real-time vibrotactile feedback on eating rate (cf. 9 for a detailed description of the height, weight and other dimensions of the 10sFork). Before each meal, participants are invited to turn on their fork, which is shown by a green indicator light on the handle. When taking a bite, the conductive surface on the fork prongs connects through the body of the user with the conductive surface of the handle; this short circuit is detected, assessed, and if it represents a bite, its timestamp is stored. The fork delivers vibrotactile feedback at a pre-set interval between bites; in this study, the interval was set to the factory default of 10 s. If users take a bite too quickly (i.e. before the end of the 10-s interval), they feel a gentle vibration in the handle of the fork and see a red indicator light. The fork stores each ‘bite’ with a unique ID and timestamp, which enables determining meal duration through the exact time at which the meal is started and ended. Furthermore, it counts the total number of bites per meal and per minute, and the average interval between bites. Finally, it measures the ratio of ‘correct’ bites versus bites within the 10s timeframe. All data is stored on the fork and can be synchronized with an online platform. In addition to the vibrotactile feedback, (only the) participants in VFC+ had access to a secure online platform, where they could review retrospective visual feedback on (trends in) meal duration, number of bites, and over-speed ratio of their past meals. A screenshot of the online dashboard is available from the project’s OSF site.

### Measures

#### Primary outcome measures

*Bite rate* and *success ratio*. For every participant, the 10sFork was set up to automatically record each bite of each individual meal. For each unique meal, participants’ *bite rate* (i.e., the average number of bites per minute ((total number of bites divided by meal duration in seconds) multiplied by 60) and *success ratio* (i.e., number of bites outside 10-s time interval divided by total bites) were calculated automatically by a script on the SlowConnect server.

*BMI.* Participants’ weight and height were measured by their dieticians, following standard procedures, at three moments: at baseline (T1), directly after the 4-week intervention period (T2) and at the follow-up after eight weeks (T3). Participants’ BMI was calculated as weight in kilograms divided by height in meters squared.

#### Secondary outcome measures

*Meal duration*. Meal duration was calculated as the time in minutes between the first and last bite of every unique meal.

*Pause duration*. The average time interval between fork servings (i.e. pause duration), was determined by calculating the average interval between bites for each unique meal.

*Total bites*. The total number of fork servings for each unique meal was calculated by a script on the SlowConnect server.

*Other measures*. We determined participants’ *age, gender, perceived eating rate at baseline,* and markers of health condition – *dietary restraint*, *diabetes I and II*, *perceived stomach complaints* (i.e., heartburn, regurgitation, bloating, obstipation, flatulence), and *discomfort during eating*, through questions taken from the online baseline survey; perceived eating rate was determined by selecting a value on a ten-point scale with 1 referring to ‘I eat slower than any other person’ and 10 referring to ‘I eat faster than any other person’. Markers of health condition were determined by digital choices (yes / no).

### Statistical analyses

We inspected the sample distributions and distributions of the mean of our primary and secondary outcome variables and their potential confounders (age, gender, perceived eating rate at baseline, health condition, and BMI at baseline), testing skewness, kurtosis, and performing Hartigan’s dip test [12], Shapiro Wilk-test for normality [13], Anderson-Darling-test for goodness of fit [14], and Kolmogorov-Smirnov test for equality of distributions [15]. We then performed randomization checks for group equivalence, calculating Cramér’s V for categorical variables, and Omega squared for continuous variables. Finally, we checked whether BMI and secondary outcome measures *– meal duration, pause duration, and total bites –* were associated at baseline with bite rate and success ratio. These data inspection analyses are available from the open data set (see below).

To assess the short- and medium-term effectiveness of the augmented fork on our primary (*bite rate, success ratio,* and *BMI*) and secondary (*meal duration, pause duration, total bites*) outcome measures over time, we performed mixed model analyses of variance with Satterthwaite degrees of freedom estimation. Participant ID was entered as a random variable, and (intervention) condition, (intervention) phase and (intervention) condition × (intervention) phase as fixed variables. Inspection analyses were done in R version 3.3.2 for MacOS X with RStudio version 1.0.136 for MacOS X [16], and multilevel analysis was done using SAS for Windows, using the PROC MIXED procedure (version 9.2).

Statistical power calculations to determine the sample size needed for multilevel modelling tend to be more complex than those needed for single-level designs, because of the statistical dependency of clustered data. Rules of thumb for sample size per cell on the lowest level range from 30 to 50 participants per cell [17, 18]. With three cells in a single level (condition), and three repeated observations per participant, we needed to include 90–150 participants in our trials to obtain 80% power to detect a medium effect size (as found in 10), with a significance level of 0.05, assuming the ratio of the variability of the level 1 coefficient to the variability of the level 1 residual is 1.

## Results

Of the total number of 163 participants, 141 were randomly allocated to one of three intervention groups: intervention conditions VFC and VFC+, and control condition NFC (see Fig. 1). Table 1 shows the baseline characteristics per intervention group. Of the 141 participants allocated to the three conditions, 17 (12.1%; VFC 4, VFC+ 6, NFC 7; technical issues: *n* = 6, lost interest / health-related issues: *n* = 11) dropped out before reaching T2, the post-measurement period directly after the intervention. At T3, a further 28 participants had dropped out (19.86%; VFC 14, VFC+ 4, NFC 10; never received fork for post-measurement: *n* = 20, lost interest / health-related issues: *n* = 8).

During the intervention period, participants ate a median number of 23 meals (range 5–79, average 27.8 meals, *SD* = 16.3) with the 10sFork in 4 weeks, an average of almost one meal per day (Table 2). The number of meals eaten with the fork during the intervention period were similar between conditions (VFC *m* = 25.6 ± 14.7; VFC+ *m* = 28.9 ± 18.0; NFC *m* = 29.7 ± 16.5, ω^2^ = .008). In Table 2, the median and range of meals for each condition are listed.

**Table 2:** Number of meals eaten with the fork: median (range)^a^

	NFC	VFC	VFC+
T1	5 (2–15)	5 (2–28)	5 (3–15)
Intervention period	24 (7–66)	23 (9–75)	23 (7–79)
T2	5 (1–14)	4 (1–14)	5 (1–17)
T3	5 (2–13)	5 (2–17)	5 (2–20)

^a^ – *NFC* no feedback condition, *VFC* vibrotactile feedback condition, *VFC+* vibrotactile + visual feedback condition

T1: baseline measurement before the intervention period; T2: follow-up measurement directly after the intervention period; T3: follow-up measurement eight weeks after the intervention period. During these five-day measurement periods, subjects were instructed to eat as many meals as possible with the 10sFork, with all feedback switched off

Of the 38 participants in the VFC+ condition, 21 used the online dashboard for additional retrospective visual feedback on their eating rate: four participants used the dashboard more than once per week, 11 used it once per week at most, 3 used it a couple of times in the 4-week intervention period, and 3 more used it only once in the intervention period.

### Primary outcome measures

The results for the effect of vibrotactile feedback on bite rate, success ratio, and BMI at baseline, at T2 (directly after the intervention period) and T3 (follow-up after eight weeks) are shown for the three different intervention groups in Table 3.

**Table 3:** Bite rate, Success Ratio, and BMI at baseline, T2^1^, and T3^1^ of the three different intervention groups with *p*–values for Phase (T1, T2, or T3), Condition (NFC, VFC, or VFC+), and Phase*Condition^*^; for number of observations see the flowchart. Mean ± SD

	NFC	VFC	VFC+	*Phase*	*Condition*	*Phase x condition* p-value*
Bite Rate (bites/min):						
Baseline	6.4 ± 0.3^a^	6.2 ± 0.3^a^	5.9 ± 0.3^a^			
T2	5.6 ± 0.3^b^	4.4 ± 0.3^b†^	4.1 ± 0.4^b†^			
T3	6.2 ± 0.4^b^	4.9 ± 0.3^b†^	4.6 ± 0.4^b†^	***.02***	***< .0001***	***< .01***
Success ratio (%):						
Baseline	45.2 ± 2.9^a^	43.2 ± 2.7^a^	45.4 ± 3.0^a^			
T2	44.3 ± 3.0^a^	65.3 ± 2.8^b†^	68.2 ± 3.1^b†^			
T3	42.2 ± 3.2^a^	55.3 ± 3.0^c†^	57.5 ± 3.1^c†^	***0.002***	***< .0001***	***< .0001***
BMI (kg/m^2^):						
Baseline	31.7 ± 0.8^a^	31.1 ± 0.7^a^	31.6 ± 0.8^a^			
T2	31.9 ± 0.8^a^	30.6 ± 0.7^b†^	31.2 ± 0.8^b†^			
T3	31.3 ± 0.8^a^	30.3 ± 0.7^c†^	30.9 ± 0.8^c†^	***< .0001***	*.64*	***.019***

1 *NFC* no feedback condition, *VFC* vibrotactile feedback condition, *VFC+* vibrotactile + visual feedback condition

T1: baseline measurement before the intervention period; T2: follow-up measurement directly after the intervention period; T3: follow-up measurement eight weeks after the intervention period

* *p*-values Mixed model ANOVA, fixed factors: phase of the study, condition, phase*condition, random factor: subject. **Boldface:** significant at at least *p* < .05

Columns with different letters are significantly different (at least *p* < 0.05)

Rows with different symbols are significantly different (at least *p* < 0.05)

#### Bite rate

We observed a significant interaction between treatment / condition (VFC, VFC+ or NFC) and phase (T1, T2, or T3, *F* (4,1886) = 3.49, *p* < .01). Participants in the experimental conditions slowed down their bite rate from 6.2 ± 0.3 (VFC) and 5.9 ± 0.3 bites/min (VFC+) at baseline (T1) to 4.4 ± 0.3 and 4.1 ± 0.4 bites/min at T2 respectively (all *p*-values for comparison with T1 < .0001). At T3, this was 4.9 ± 0.3 and 4.6 ± 0.4 bites/min for VFC and VFC+, respectively (all *p*-values for comparison with T1 < .0001). Participants in the control condition (NFC) also managed to slow down their bite rate; from 6.4 ± 0.3 bites/min at baseline (T1 to 5.6 ± 0.3 at T2 (comparison with T1 *p* < .05). No further deceleration of bite rate was shown at T3, that is 6.2 ± 0.4 (comparison with T1 *p* = .33). The effect size (Cohen’s *d*) for the interaction between phase and condition was 0.346.

#### Success ratio

We found significant main effects of vibrotactile feedback on success ratio, i.e. the number of bites with a 10-s pause between them, for both condition (NFC, VFC or VFC+, *F* (2, 124) = 6.55, *p* = .002) and phase (T1, T2 or T3, *F* (2, 1888) = 72.51, *p* < .0001). These main effects were qualified by a significant interaction between condition and phase (*F* (4, 1887) = 21.05, *p* < .0001). Participants in the experimental condition improved their success rate; those in VFC went from 43.2% (*SE* = 2.7) at baseline to 65.3% (*SE* = 2.8) at T2 and 55.3% (*SE* = 3.0) at T3 (all *p* < .0001 for comparisons with T1); participants in VFC+ went from 45.4 (*SE* = 3.0) at baseline to 68.2% (*SD* = 3.1) at T2 and 57.5 (*SD* = 3.1) at T3 (all *p* < .0001 for comparisons with T1). Participants in the control condition did not improve their success rate. The effect size (Cohen’s *d*) for the interaction between phase and condition was 0.89.

#### BMI

We found a significant main effect of the vibrotactile feedback on BMI for phase (T1, T2 or T3, *F* (2, 213) = 19.11, *p* < .0001). However, this effect was qualified by a significant interaction between phase and condition (NFC, VFC or VFC+, *F* (4, 213) = 3.00, *p* = .02).

On average, participants in the experimental conditions lost weight during the intervention period: going from 31.1 (VFC, *SD* = 0.7, *t* = 3.24, *p* < .001) and 31.6 (VFC+, *SD* = 0.8, *t* = 2.43, *p* < .02) kg/m^2^ at baseline to 30.6 (VFC, *SD* = 0.7) and 31.2 (VFC+, *SD* = 0.8) kg/m^2^ at T2. On average, participants in the control condition did not lose weight during the intervention period, going from 31.3 kg/m^2^ (*SE* = 0.8) at baseline to 31.9 kg/m^2^ (*SE* = 0.8) at T2, *t* = − 1.34, *p* = .18. Participants in all conditions lost weight after the intervention period, with average BMIs of 31.3 (NFC, *SD* = 0.8, t = 2.12, *p* < .05), 30.3 (VFC, *SD* = 0.7, *t* = 5.09, *p* < .0001) and 30.9 (VFC+, *SD* = 0.8 m *t* = 3.56, *p* < .001) kg/m^2^ at T3 (comparisons with T1). The effect size (Cohen’s *d*) for the interaction between phase and condition was 0.34.

### Secondary outcome measures

We found a main effect of the vibrotactile feedback on *meal duration* for phase (T1, T2, or T3, *F* (2, 1905) = 11.86, *p* < .0001). There was no significant interaction between phase and condition (*F* (4.1903) = 1.72, *p* = .14). All participants spent more time on their meals at T2 (NFC: 683 ± 38, VFC: 722 ± 35, VFC+: 679 ± 38 s) and T3 (NFC: 683 ± 40, VFC: 752 ± 38, VFC+: 791 ± 38 s) than at baseline (NFC: 589 ± 35, VFC: 694 ± 34, and VFC+: 651 ± 37 s). See Table 4 for a full overview of multilevel analyses of all primary outcome measures.

**Table 4:** Meal duration, Total bites per meal, and Pause duration at baseline, T2^1^, and T3^1^ of the three different intervention groups with *p* - values for Phase (T1, T2, or T3), Condition (NFC, VFC, or VFC+), and Phase*Condition^*^; for number of observations see the flowchart. Mean ± SD

	NFC	VFC	VFC+	*Phase*	*Condition*	*Phase x condition*
Meal duration (seconds):						
Baseline	589 ± 35^a^	694 ± 34^†^	651 ± 37^a†^			
T2	683 ± 38^b^	722 ± 35	679 ± 38^a^			
T3	683 ± 40^b^	752 ± 38	791 ± 38^b^	***< .0001***	*.24*	*.14*
Total bites (per meal):						
Baseline	55.8 ± 4.1	65.5 ± 3.9	59.2 ± 4.2			
T2	59.8 ± 4.2^a†^	49.9 ± 3.9^ab†^	41.9 ± 4.3^b†^			
T3	63.8 ± 4.4^†^	57.3 ± 4.2	53.3 ± 4.3	***< .0001***	*.29*	***< .0001***
Pause duration (seconds):						
Baseline	13.1 ± 1.1^a^	13.1 ± 1.1^a^	14.3 ± 1.2^a^			
T2	14.7 ± 1.2^a^	19.3 ± 1.1^b†^	21.3 ± 1.2^b†^			
T3	15.2 ± 1.3^a†^	17.7 ± 1.3^b†‡^	19.8 ± 1.2^b‡^	***< .0001***	*.64*	***.019***

1 *NFC* no feedback condition, *VFC* vibrotactile feedback condition, *VFC+* vibrotactile + visual feedback condition

T1: Baseline measurement before the intervention period; T2: follow-up measurement directly after the intervention period; T3: follow-up measurement eight weeks after the intervention period.

* *p*-values Mixed model ANOVA, fixed factors: phase of the study, condition, phase*condition, random factor: subject. **Boldface:** significant at at least *p* < .05

Columns with different letters are significantly different (at least *p* < 0.05)

Rows with different symbols are significantly different (at least *p* < 0.05)

We found a main effect of the vibrotactile feedback on *total number of bites per meal* for phase (T1, T2, or T3, *F* (2, 1882) = 17.74, *p* < .0001), which was qualified by an interaction effect of phase (T1, T2, or T3) and condition (NFC, VFC, or VFC+, *F* (4, 1881) = 9.12, *p* < .0001); participants in the experimental conditions took less bites per meal at T2 and T3 when compared to the baseline, whereas participants in the control condition NFC took more bites per meal when compared to the baseline (see Table 4).

We found a main effect of the vibrotactile feedback on *pause duration* for both phase (T1, T2, or T3, *F* (2, 129) = 4.04, *p* = 0.02) and condition (NFC, VFC, or VFC+, *F* (2, 1915) = 36.23, *p* < .0001) which was qualified by an interaction between phase and condition (*F* (4, 1913) = 3.75, *p* < .01). All participants took longer pauses between bites, but the prolongation was limited to (on average) 1.6 s at T2 and (on average) 2.1 s at T3 for the control condition NFC, whereas the experimental conditions managed an average prolongation of 6.2 s at T2 and 4.6 s at T3 for VFC, and 7 s at T2 and 5.5 s at T3 for VFC+ (see Table 4).

## Discussion

The present study assessed the effects of a technology-based feedback intervention on eating rate and body weight in naturalistic eating contexts. We examined the effects of vibrotactile and retrospective visual feedback on participants’ eating rate and body weight over a 15-week period. To do so, we conducted a three-armed parallel group RCT with two measures of eating rate (i.e., bite rate and success ratio) and body weight, measured at baseline (T1), directly after a 4-week intervention period (post-intervention, T2) and eight weeks later (follow-up, T3).

Our findings showed that technology-based vibrotactile feedback helps to sustainably slow down eating rate. The vibrotactile feedback affected both bite rate, i.e. the total average of bites per minute during the entire meal, and success ratio, i.e. the ‘spacing’ of bites during a meal. After four weeks using the fork with vibrotactile feedback, participants in the experimental conditions slowed their eating rate by an average of 1.8 bites per minute (20–25%), and improved their success ratio, the number of bites with at least a ten-second pause between them, by an average of 22.5%. This suggests that the vibrotactile feedback delivered by the fork teaches people to slow down their ‘fast’ bites. Our findings on pause duration confirm this pattern: participants in the intervention conditions had significantly longer pauses between bites. This is in line with our hypothesis that people who received concurrent vibrotactile feedback on eating rate would succeed in eating more slowly, with both a lower average number of bites per minute (bite rate), and more time between bites (success ratio). Our results confirm and extend findings from a previous (lab) study with the 10sFork [10], in which vibrotactile feedback from the fork had a significant effect on both bite rate and success ratio in a single meal, but with the latter effect much more pronounced: effect sizes (Cohen’s *d* [19]) showed a small to moderate effect of the vibrotactile feedback on bite rate, whereas effect sizes for success ratio point at a moderate to large effect of vibrotactile feedback on success ratio. The finding that the effects of bite rate and success ratio remained significant for the experimental conditions at an eight-week follow-up measurement shows that feedback from digital technology has the potential to bring about long-lasting changes in eating rate.

All participants, regardless of condition, had longer meals. This may very well be an effect of the demand characteristics of eating with the 10sFork, which in itself encourages more mindful eating. This is in line with previous findings [9] where people reported that eating with the fork made them more aware of their eating behavior. Mindful eating and the demand characteristics of using the 10sFork might also explain why participants in the control condition (NFC) managed to slightly slow down their bite rate directly after the intervention (at T2) without vibrotactile feedback. However, participants in the experimental conditions (VFC and VFC+) showed a stronger deceleration than those in NFC. Furthermore, only in the intervention groups did eating with the fork lead to a higher success ratio, longer pauses between bites, and less bites per meal. Finally, the deceleration of eating rate directly after the intervention (T2) did not remain stable for the control condition: at the eight-week follow-up measurement (T3), participants in NFC no longer showed lower bite rates when compared to the baseline measurements, whereas participants in the experimental conditions still ate at a lower bite rate and with a higher success ratio at the eight-week follow-up.

With regard to participants’ body weight, we found that participants in the intervention (VFC and VFC+) groups managed to lose weight, whereas participants in the control group did not. At the follow-up measurement after eight weeks, participants in both intervention groups had not regained their lost body weight. This finding is in line with other feedback-based interventions to reduce eating rate, which, in clinical settings, also found durable weight loss among adults [20, 21] and children [22]. Yet, participants in the control condition managed to also lose weight in the period between the first follow-up measurement directly after the intervention period (T2) and the follow-up measurement after eight weeks (T3). This may be due to the fact that all participants were visiting a dietician, which may have helped participants to lose some weight in this condition as well. More confirmatory research is needed to shed light on the sustainability of the weight loss obtained by eating with the fork and the effect of training duration on weight loss.

Our finding that vibrotactile feedback on eating rate leads to weight loss may be explained by a number of potential underlying mechanisms. The fact that participants in the intervention groups took fewer bites per meal may have meant that they also ate smaller portions, and consequently less calories. However, this is speculation and needs to be investigated further. Eating rate may affect body weight through multiple (biological) mechanisms such as changes in the secretion of satiety hormones [23, 24]; lower energy intake through enhanced oral exposure [25, 26] and higher number of chews per unit of food [27, 28]; decreased feelings of deprivation by enhancing and prolonging pleasurable aspects of eating [29]; and changes in the encoding of the meal in memory, which in turn influences food choices in subsequent meals [30, 31]. Finally, the vibrotactile feedback may have made people more aware of their goal to lose weight or eat less, which may have strengthened their resolve in other nutrition-related behaviors than eating rate, such as eating healthier meals [8]. Further research in this domain is needed to gain insight into which (combination of) underlying mechanisms caused the reduction in BMI.

While we found clear differences in eating rate (bite rate and success ratio) and BMI between the experimental conditions VFC and VFC+ on one hand, and the control condition NFC on the other, we found no differences between VFC and VFC+ in any measure. This shows that the retrospective visual feedback had no (further) effect on eating rate nor BMI. Evidence [7], however, suggests that this kind of visual feedback would serve to motivate adherence to the intervention, and strengthen the effects of the vibrotactile feedback on our primary outcome measures. One reason of the lack of success of the visual feedback may lay in the relatively low uptake of the visual feedback. Only 21 of the 39 participants in VFC+ used the dashboard, and of those 21, 17 participants used it at most once a week. Thus, the lack of effect of the visual feedback may be due to a lack of engagement with the dashboard. Further research into the user experience and efficacy of the dashboard environment is needed to examine the potential effects of visual feedback on motivation adherence and/or eating rate.

An important strength of this study was the assessment of the effectiveness of technology-based feedback on eating rate and body weight in naturalistic eating contexts. Our study contributes to a stronger empirical base for feedback-based interventions delivered through digital technology in altering deeply engrained habitual behaviors. Despite these strengths, a few limitations should be mentioned. First, there was a drop-out level of 12.1% (*n* = 17) during the intervention phase, and a further drop-out of 19.8% [23] at the eight-week follow-up. Although this level of drop-out is not uncommon for field trials, we cannot rule out the possibility that bias from drop-out could have affected our results. It is worth mentioning that most drop-outs were unrelated to the intervention, but were mostly caused by technical issues or forks being mislaid in the mail. Further, these dropouts were equally distributed among conditions. Second, we could not include daily dietary or nutritional intake measures in our study, as this would have put a high burden on individuals participating in our study. Repeatedly asking participants for the type and amount of food consumed while using the fork, would have induced a demand characteristic that would have interfered with the ‘natural’ use of the fork in the intervention phase [32], compromising the validity of our findings. As a result, however, we do not have an indication of the type and amount of food people ate nor do we have a reliable measurement of individuals’ satiety levels after the meal. Although the specific aim of the present study was *not* to examine how fork use would affect food intake, further research with more exhaustive registrations of participants’ meals and satiety can help to better understand how a lower eating rate leads to weight loss. In a similar vein, we have no information about the number of meals or in-between meal snacks eaten without the fork. Participants in all conditions were free to eat snacks and other foods with their hands or with other, non-smart cutlery. However, we believe that this does not reduce the value of the results of our study. On the contrary, even if participants had the opportunity to derail the effects of the training by consuming snacks and meals without the fork, the effect was still observed. Third, at baseline, there seems to be a high occurrence of stomach problems (with 47–55% of the participants reporting some sort of stomach problem). However, we believe this has to do with the wording of the question about stomach problems in the first questionnaire, in which participants score ‘yes’ on perceived stomach problems regardless of quantity, severity and kind of problem (flatulence, heartburn, regurgitation, bloated feeling). Future research could examine which stomach problems occur and their severity and frequency. Fortunately, there was no effect of stomach problems on eating rate and success ratio at baseline, and no differences between experimental conditions appeared.

Our findings have direct implications for the clinical management of weight control, highlighting the importance of a slow eating rate in addition to more traditional dietary instructions on what and how much to eat. The results of the present study suggest that technology-based vibrotactile feedback provides dieticians and other care professionals with an effective intervention to slow down eating rate. Until now, there was no feasible and easy-to-use intervention available to address eating rate in the home setting. The 10sFork is a promising development in this area. This intervention, combined with other interventions aimed at healthier eating and increased physical activity, may further help dieticians in supporting clients in achieving lasting weight loss, reducing physical complaints, and in the prevention and perhaps even the treatment of debilitating conditions such as diabetes type II [33].

## Conclusion

This study showed that vibrotactile feedback from an augmented fork to decrease eating rate could be an effective tool to reduce eating rate. After a 4-week intervention period, people who received vibrotactile feedback on eating rate managed to achieve longer spacing between bites, leading to fewer fast bites, longer pauses between bites, less bites per meal, and significant weight loss. Our results indicate that these effects remain after an eight-week pause in feedback suggesting that changes in eating rate as a result of using the fork may be durable. Further research is needed to shed light on longer-term efficacy of this intervention on eating rate and body weight.

## Data Availability

The dataset supporting the conclusions of this article is available in the EASY data repository system of DANS, the Netherlands Institute for Permanent Access to Digital Research Resources, 10.17026/dans-zqw-6fca. All instruction materials for the study are available from the Take It Slow OSF site at https://osf.io/753nf/.
